# Thermal Liquid Biopsy (TLB): A Predictive Score Derived from Serum Thermograms as a Clinical Tool for Screening Lung Cancer Patients

**DOI:** 10.3390/cancers11071012

**Published:** 2019-07-19

**Authors:** Alberto Rodrigo, Jorge L. Ojeda, Sonia Vega, Oscar Sanchez-Gracia, Angel Lanas, Dolores Isla, Adrian Velazquez-Campoy, Olga Abian

**Affiliations:** 1 Medical Oncology Department, Hospital Arnau de Vilanova, 25198 Lleida, Spain; 2 Department of Statistical Methods, Universidad de Zaragoza, 50009 Zaragoza, Spain; 3 Institute of Biocomputation and Physics of Complex Systems (BIFI), Joint Units IQFR-CSIC-BIFI, and GBsC-CSIC-BIFI, Universidad de Zaragoza, 50018 Zaragoza, Spain; 4 Enrique Val, 50011 Zaragoza, Spain; 5 Department of Medicine, University of Zaragoza, 50009 Zaragoza, Spain; 6 Aragon Institute for Health Research (IIS-Aragon), 50009 Zaragoza, Spain; 7 Servicio de Aparato Digestivo, Hospital Clinico Universitario Lozano Blesa, 50009 Zaragoza, Spain; 8 Biomedical Research Networking Center in Digestive and Hepatic Diseases (CIBERehd), 28029 Madrid, Spain; 9 Medical Oncology Department, Hospital Clinico Universitario Lozano Blesa, 50009 Zaragoza, Spain; 10 Department of Biochemistry and Molecular and Cell Biology, Universidad de Zaragoza, 50009 Zaragoza, Spain; 11 Fundacion ARAID, Government of Aragon, 50018 Zaragoza, Spain; 12 Aragon Health Sciences Institute (IACS), 50009 Zaragoza, Spain

**Keywords:** liquid biopsy, lung cancer, serum sample, differential scanning calorimetry, generalized linear models, cancer screening program

## Abstract

Risk population screening programs are instrumental for advancing cancer management and reducing economic costs of therapeutic interventions and the burden of the disease, as well as increasing the survival rate and improving the quality of life for cancer patients. Lung cancer, with high incidence and mortality rates, is not excluded from this situation. The success of screening programs relies on many factors, with some of them being the appropriate definition of the risk population and the implementation of detection techniques with an optimal discrimination power and strong patient adherence. Liquid biopsy based on serum or plasma detection of circulating tumor cells or DNA/RNA is increasingly employed nowadays, but certain limitations constrain its wide application. In this work, we present a new implementation of thermal liquid biopsy (TLB) for lung cancer patients. TLB provides a prediction score based on the ability to detect plasma/serum proteome alterations through calorimetric thermograms that strongly correlates with the presence of lung cancer disease (91% accuracy rate, 90% sensitivity, 92% specificity, diagnostic odds ratio 104). TLB is a quick, minimally-invasive, low-risk technique that can be applied in clinical practice for evidencing lung cancer, and it can be used in screening and monitoring actions.

## 1. Introduction

### 1.1. Liquid Biopsy (LB)

Solid tumors exhibit remarkable complexity, with large cellular heterogeneity, where different types of cells (cancer cells, fibroblasts, adipocytes, endothelial cells, immune cells, and the extracellular matrix (ECM)) coexist [1,2]. All these components extensively interact and are responsible for cancer development, suggesting that cancer cells are not completely autonomous. The development of cancer strongly depends on the interplay of surrounding cells and non-cellular components which are present in the microenvironment. All this is crucial for establishing an appropriate niche for the tumor to develop in, regulating its growth, and allowing cancer cells to evolve. Therefore, the interaction between tumor cells and the microenvironment is the basis for the development, progression, and therapy response in solid tumors [3].

The ECM is constantly being remodeled; its cells display constant activity, which in turn depends to a large extent on the extracellular matrix itself [2]. The result of this remodeling process is the release of different ECM components into the bloodstream, which represents the main source for a variety of biomarkers reporting on tumor characteristics, growth, and development [4,5].

Liquid biopsy (LB) is a technology based on the sampling and analysis of non-solid biological tissue for the diagnosis, prognosis, and prediction of a therapeutic response of a certain illness, without the need for costly or more invasive procedures. The interest of LB clinical implementation for cancer diagnosis has recently increased and many bibliographical references on this topic can be found [6,7,8].

The optimal application of LB in clinical practice requires solving some aspects that may limit its implementation. Some aspects are related to standardization of the pre-analytical and analytical process; for example, the sample should be subjected to as little manipulation as possible and its preservation must be controlled in order to guarantee the stability of the biomarkers of interest [9]. Other aspects are related to the expected high sensitivity for the analytical procedure in order to be used in the early detection of cancer, allowing small tumors, undetectable by conventional imaging techniques, to be identified [10,11]. Finally, specific biomarkers for each type of cancer must be identified and quantified [12]. Efforts of researchers and companies developing LB approaches are focused on these aspects [6,7].

Several major obstacles complicate the application and interpretation of LB in clinical practice, as follows: (1) mapping of tumor biochemical, histological, and immunological features and LB parameters for obtaining information on a potential tumor; (2) handling and measuring of the biological sample; (3) obtaining enough of a sample for measurement; (4) identifying specific and non-specific tumor-related biomarkers for patient classification and prioritization; and (5) cell population heterogeneity in solid tumors.

### 1.2. Thermal Liquid Biopsy (TLB)

In the last decade, differential scanning calorimetry (DSC) analysis of blood plasma has arisen as a promising approach for the discrimination of healthy and cancer patients [13,14,15,16]. A DSC thermogram reflects the global denaturation profile for all major blood plasma proteins and their potential interactions with other blood plasma constituents (proteins and metabolites). The composition of blood serum can be significantly altered, either by the up- or down-regulation of proteins, or by the release of disease-related metabolites into the bloodstream. A plasma thermogram would reflect the distortion caused by the tumor. Therefore, this technique could be employed as a clinical test for diagnosing, classifying, and monitoring patients, since the thermograms from a diseased subject and a healthy subject can be considerably different [17,18]. Our group has previously shown that DSC can be applied for classifying and staging gastric adenocarcinoma patients and monitoring melanoma patients during their treatment [19]. 

These previous works represent the proof of principle for implementing what we have termed “thermal liquid biopsy” (TLB, i.e., DSC thermogram analysis of a serum sample) as a valuable approach for a personalized diagnostic assessment of cancer patients. This methodology would provide physicians with a closer and personalized monitoring protocol to help in assessing patient health status and to facilitate the decision-making process. Combining thermograms with a multiparametric analysis allows for definitive single diagnostic values to be obtained, which can then be compared with the control reference values. The provided score is a measure of the alteration of the blood plasma proteome, which, as reported here, shows a strong correlation with the presence of lung cancer. TLB can be regarded as a minimally-invasive, low risk, quick technique complementary to current imaging modalities, establishing congruent surveillance schemes by performing routine imaging as currently prescribed (e.g., 2–4 times a year), while proposing more frequent TLB tests (e.g., monthly or bimonthly).

### 1.3. Lung Cancer

According to the Global Cancer Observatory data [20], lung cancer ranked first in 2018 as the cancer with the highest worldwide incidence and mortality rate in men and the second in women (more deaths than colon, breast, and prostate cancers combined). This represents a total of 2 million new cases and 1.7 million deaths in 2018.

Successful therapeutic treatment or surgical intervention of the tumor require appropriate and accurate disease prognosis and early stage detection (I−IIIB); however, a lack of relapse after removal of the tumor or absence of metastasis cannot be guaranteed. Different actions have been implemented as a means of reducing the social and health impact of lung cancer, as follows: (1) prevention campaigns leading to a reduction in tobacco consumption; and (2) screening programs in risk populations for reducing lung cancer mortality by detecting the disease in its early stages, when tumors can be surgically resected or treated with chemotherapy with a better prognosis.

Among the imaging techniques employed for lung cancer identification and characterization, low-dose computed tomography (LDCT) was introduced in a United States National Lung Screening Trial, achieving a 20% reduction in specific mortality. This study showed that LDCT had a greater sensitivity than a chest X-ray for lung cancer diagnosis [21]. However, this screening protocol based on LDCT presented a series of severe drawbacks, such as the high false positive rate, uncertainty about the suitable periodicity between follow-up tests, and the cost-effectiveness of the protocol in individuals at a high risk of suffering from lung cancer [22].

In order to improve the cost-effectiveness of the test, studies have been carried out to define, in a very precise way, the selection criteria for the risk population which the screening is aimed at, and which subpopulation would benefit the most from it [23,24]. Epidemiological factors must be taken into account, in addition to age and tobacco consumption.

In this sense, TLB is presented as a complementary method that, combined with imaging techniques, can serve to improve the sensitivity and specificity of the screening method if adopted in clinical practice [25,26,27].

### 1.4. TLB for Lung Cancer Diagnosis and Monitoring

Compared to current technological procedures for inferring information on the physiological characteristics of tumors, including liquid biopsy based on circulating tumor cells and DNA, the advantages of TLB are the following:

(1) Simple and quick sample handling: once serum from the blood sample is obtained, it can be stored, frozen, and TLB-measured by simple dilution in a phosphate-buffered saline solution after thawing (when stored at −80 °C, samples remain after several freezing/thawing cycles);
(2) High sensitivity measurement: TLB is able to detect changes in the serum composition (down- or upregulation of major serum proteins, presence of interacting tumor-related metabolites, tumor-related posttranslational modification of major serum proteins) at early stages of the illness, even when lesions are not visible using imaging techniques [18];
(3) Broad clinical scope: TLB can be applied to different types of cancers, since the analytical detection method is based on real-time observation of the tumor-induced altered metabolic state of the serum of a given subject compared to that of healthy subjects (that is, TLB does not detect any specific tumor-related component);
(4) Quasi-real-time monitoring: TLB assesses the current proteome serum health status, instead of providing a cancer risk evaluation based on genetic biomarkers.

TLB can be used to boost the adherence of patients to surveillance actions in a given population and to increase the frequency of evaluation procedures by reducing the monitoring time interval without a significant increase in economic cost or burden/risk for the patient, while providing diagnostic value for a patient's current disease state. A high positive association between clinical groups and TLB assessment offers advantages over current diagnosis techniques (e.g., LDCT imaging), providing a powerful diagnostic and monitoring approach with a minimally-invasive, low-risk, low-cost clinical test for lung cancer. Promising future applications, such as implementation within screening programs, could be developed from TLB. As a first step towards this goal, our main objective in this work is to determine the ability of TLB to discriminate between healthy controls (HC) and lung cancer patients (LCP).

## 2. Results

### 2.1. TLB Serum Thermograms for HC and LCP Individuals

**Figure 1:**
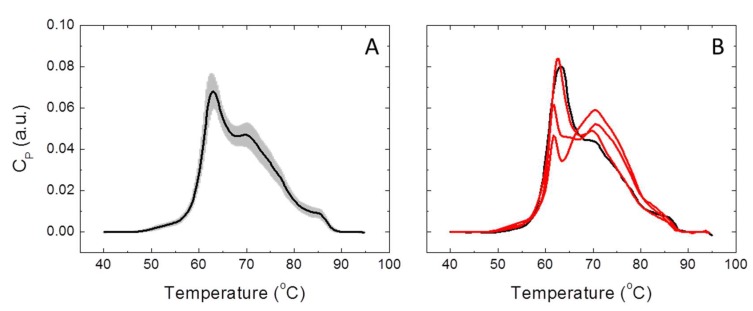
Thermal liquid biopsy (TLB) serum thermograms for healthy control (HC) and lung cancer patient (LCP) individuals. (**A**) Average TLB thermogram calculated with all subjects belonging to the HC group (continuous line) is shown together with the standard deviation of the thermograms at each temperature (shaded region). (**B**) TLB thermogram for a healthy subject (black line) compared to three thermograms from lung cancer patients (red lines).

Serum thermograms were measured for each individual belonging to HC (*n* = 85) and LCP (*n* = 114) groups. Figure 1 shows representative thermograms for healthy and diseased subjects. The homogeneity of serum thermograms within the HC group, as well as their heterogeneity within the LCP group, is remarkable. Moreover, significant differences in the TLB thermograms of healthy and diseased subjects are apparent. The application of the deconvolution procedure (see Materials and Methods) provides the multiparametric set representing the basis for the statistical methodology for classifying and discriminating between HC and LCP individuals.

### 2.2. Analysis of Individual TLB-Derived Parameters

As a preliminary statistical evaluation of the ability of the different parameters to classify individuals into HC and LCP groups, we determined the main quartiles Q_1_, Q_2_, and Q_3_ (that is, the median and the first and third quartiles) for the distribution of each TLB-derived parameter within these two groups. Table 1 shows these statistical indexes for HC and LCP groups (see also Table S1 and Figure S1).

**Figure 2:**
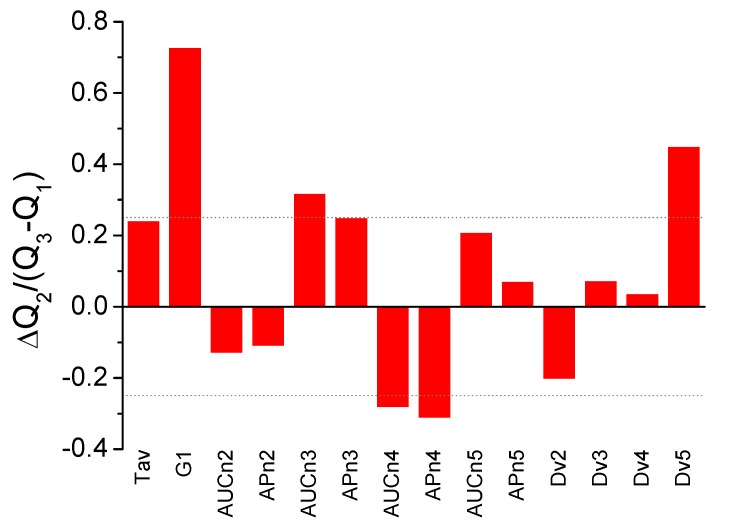
Adapted Cohen d index as a measure of size effect between healthy control (HC) and lung cancer patient (LCP) groups (difference between the median values normalized by the pooled interquartile range) for the individual parameters derived from TLB serum thermograms. Some of the parameters show large differences between the two groups: T_av_, G_1_, AUC_n3_, AP_n3_, AUC_n4_, AP_n4_, and Dv_5_ (a threshold value of ±0.25 is indicated by the dotted line).

In order to visually inspect and explore single-parameter dissimilarities between HC and LCP groups, size effects were estimated through an adapted Cohen d index, calculated as the difference between the median values normalized by the pooled (weighted-average) interquartile range for each parameter (that is, median values and interquartile ranges were employed, instead of average values and standard deviations). Therefore, not only the difference in the median for each parameter is evaluated, but also how large this difference is compared with the parameter dispersion. Figure 2 shows the normalized median difference for each parameter, and seven parameters (T_av_, G_1_, AUC_n3_, AP_n3_, AUC_n4_, AP_n4_, and Dv_5_) emerge individually as the statistically most important parameters for distinguishing both groups.

To evaluate the predictive capabilities of each of the TLB-derived parameters, we performed a Receiver Operating Characteristic (ROC) curve analysis. The main idea behind ROC analysis is the identification of an optimal cut-off value for each of the parameters that might be employed for classifying subjects as healthy or diseased, by simultaneously maximizing the statistical sensitivity and specificity. To establish the optimal cut-off, we have considered the Youden method, one of the most common methods for ROC optimization [28]. Table 2 shows the result of applying the Youden method to the HC and LCP groups. The success rate or accuracy is the probability of correctly classifying subjects as healthy (unaltered TLB thermogram and belonging to the HC group) or diseased (altered TLB thermogram and belonging to the LCP group), while the sensitivity and the specificity are the percentage of true positives (positive according to TLB and belonging to the LCP group) and true negatives (negative according to TLB and belonging to the HC group), taking their respective LCP and HC groups as a reference.

From Table 2, it is apparent that G_1_, AUC_n3_, AP_n3_, AUC_n5_, and Dv_5_ are the most successful individual parameters (high success rate, sensitivity and specificity values) in correctly classifying the subjects as healthy or diseased according to individual ROC analysis.

### 2.3. Constructing a Predictive/Classifying Score for Lung Cancer Condition with GLM

Although some of the individual TLB-derived parameters exhibit a good performance regarding subject classification, the combined use of several parameters (as suggested from the fact that the two preliminary approaches have provided slightly different subsets of relevant parameters) might significantly improve the performance of the classification score if the goal is applying TLB in clinical practice. Three models, with each using a different set of parameters, were considered. Model 1 was constructed based on T_av_, G_1_, AUC_ni_, and AP_ni_; model 2 was constructed based on Dv_i_; and model 3 considered all 14 parameters.

We conducted a comparative analysis of four tools: Binomial Generalized Linear Model with Logistic Regression (GLM), Linear Discrimination Analysis (LDA), Support-Vector Machine for classification (SVM), and Näive Bayes Classifier (NBC). The performance of these four tools was as follows: GLM > SVM > LDA > NBC, though SVM and GLM were always very close (Table S2). As is shown in the Supplementary Materials, GLM and SVM perform similarly with model 3, which is apparently the best model. In addition, the interpretation of the predictive model with SVM is not as straightforward as that of the model with GLM. For these reasons, we will only consider the GLM framework.

A comparison of the three models (1–3) was conducted using GLM. Because the Dv_i_ parameters are non-linear functions of other individual TLB-derived parameters, establishing whether or not model 3 (all 14 individual parameters) represents a significant improvement for predicting a health/disease condition was a required step.

When GLM is applied to model 1, only the individual parameters T_av_, G_1_, and AUC_n3_ have statistical significance (*p*-values < 0.05). The results of this analysis are shown in Table 3. The z-value is the regression coefficient divided by the standard error obtained by GLM. As a rule of thumb, a cut-off value of 2 (which approximately corresponds to a two-sided hypothesis test with a significance level of α = 0.05) indicates whether the corresponding parameters are important (if the absolute value of the *z*-value > 2, then its corresponding GLM coefficient is non-negligible, i.e., not null, and the parameter is important and meaningful for predicting the presence/absence of lung cancer). On the other hand, when GLM is applied to model 2, only the individual parameters Dv_3_ and Dv_5_ have statistical significance (*p*-values < 0.05). Finally, when GLM is applied to model 3, the parameters showing statistical significance are mainly the ones that were meaningful in the previous models: T_av_, G_1_, AUC_n3_, AUC_n4_, AP_n4_, AUC_n5_, and Dv_4_. Interestingly, this set of parameters does not exactly coincide with the set previously identified by the preliminary statistical analysis (Table 1 and Table 2, Figure 2).

Because the three models (1–3) apparently perform differently, we need to establish which one should be finally employed. The model equivalence tests based on the likelihood ratio (Table 4) show that model 1 is statistically equivalent to the complete model. In addition, Bayesian and Akaike Information Criteria confirm that model 1 and model 3 are rather similar.

If we compare the performance of the three models to predict lung cancer for the individuals within HC and LCP groups, as shown in Table 5, model 3 performs better than the other two models in terms of the success rate, sensitivity, and specificity (Table 5). False negatives are lung cancer patients with predicted probability score (PS) scores higher than 0.5, while false positives are healthy individuals with predicted PS scores lower than 0.5. The sensitivity and specificity are defined as the true positive and true negative rates considering the LCP group and HC group, respectively, as a reference. The diagnostic odds ratio of models 1, 2, and 3 are 54, 5, and 104, respectively.

The predictions used to build the previous table were made for those same individuals that were used to fit the model. In order to reduce any possible bias, we carried out a leave-one-out (LOO) study to get a more realistic idea of the performance of the proposed models (Table 5). In this LOO study, the probability of being healthy/diseased for an individual is calculated by fitting the model with all the data except that specific subject. While LOO produces as many sets of fitted parameters and as many PS score predictions as the dataset size (total number of HC and LCP individuals), we focus on this PS score to predict the subject condition and, then, we compute the overall success rate, the sensitivity, and the specificity. This is a type of cross-validation equivalent to considering each subject as a testing subject, while using all other subjects as a training set to make the predictions.

As a fixed set of parameters is required to define the complete model, we will only consider model 3 based on the complete set of TLB-derived parameters in order to calculate the probability (PS score) of an individual being healthy or suffering from lung cancer given the TLB thermogram. It must be emphasized that this probability, as a diagnostic tool, has to be understood as an assessment index: the smaller the PS score (closer to 0) for an individual, the higher the probability the given subject suffers from lung cancer; the larger the PS score (closer to 1) for an individual, the higher the probability the individual is healthy. It is important to emphasize that the PS score is not the probability of being healthy or suffering from lung cancer, but the probability of having an unaltered/altered protein serum TLB thermogram; an altered TLB thermogram might be induced or caused by lung cancer. That correlation between a lung cancer condition and altered TLB thermogram is supported by the good performance of our model in classifying and discriminating HC and LCP groups.

It is clear that the complete model (model 3) is the best one, achieving a 91% success rate in correctly classifying individuals, 8% false positive rate (i.e., classifying as diseased a healthy subject), and 10% false negatives (i.e., classifying as healthy a diseased subject). Even though not all TLB-derived parameters seem to be statistically significant regarding the ability to classify individuals, we decided to keep all parameters in the model (that is, using model 3).

**Figure 3:**
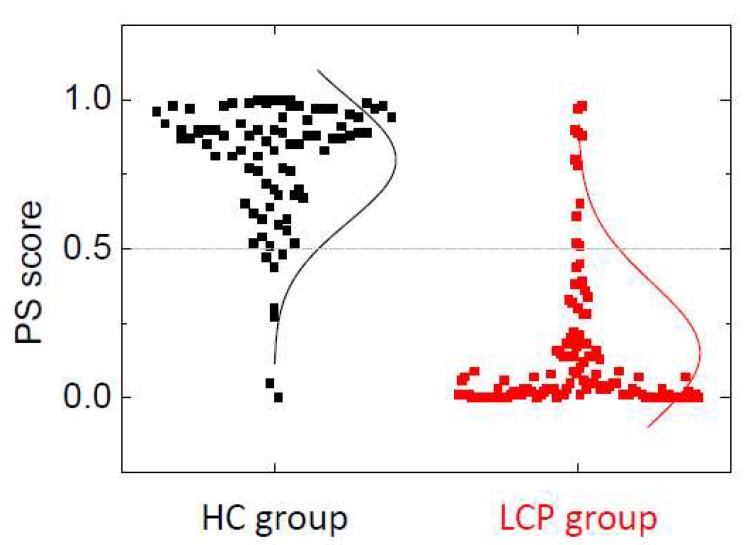
Distribution of the probability score (PS) score within healthy individuals (HC group) and lung cancer patients (LCP group). The lines represent an equivalent Gaussian distribution. The PS score threshold for discriminating between an unaltered (associated with healthy status) and altered (associated with lung cancer status) serum proteome thermal liquid biopsy (TLB) thermogram is 0.5 (grey dotted line). It can be observed that seven healthy subjects are assigned a PS score lower than 0.5 (8% false positive rate) and 11 lung cancer subjects are assigned a PS score higher than 0.5 (10% false positive rate).

Figure 3 shows the distribution of the PS score calculated with model 3 using GLM. There is a marked difference in the distribution of the PS score for HC and LCP groups: in HC, the median PS score is Q_2_ = 0.88 (Q_1_ = 0.70, Q_3_ = 0.94), while in LCP, the median PS score is Q_2_ = 0.05 (Q_1_ = 0.01, Q_3_ = 0.18). This provides a visual assessment for the quality and performance of the discriminating power of the PS score and the strong correlation between the PS score (ability to identify an unaltered/altered serum TLB thermogram) and the absence/presence of lung cancer. The distributions of PS for HC and LCP groups are very different; the difference in medians for those groups is statistically significant (*p* < 10^−15^). According to these data, 75% of the LCP subjects have a PS score below 0.18, while 75% of the HC subjects have a PS score above 0.70, resulting in an effective PS score distance of 0.52 between these two groups. Similarly, because the medians for the HC group and LCP group are 0.88 and 0.05, respectively, the difference in PS score between the 50% of HC subjects with the highest PS score (i.e., less altered TLB thermogram) and the 50% of LCP subjects with the lowest PS score (more altered TLB thermogram) is larger than 0.8. This gives an idea of the potential of the PS score for discriminating between HC and LCP subjects.

The aim of the next sections is to explore the properties of the PS score (probability of having an unaltered/altered TLB thermogram, which is related to being healthy or suffering from lung cancer) through a detailed analysis of the PS score applied to HC and LCP groups. We studied the values of the PS score according to the information available for the individuals on either their clinical history or condition in both HC and LCP groups of individuals. Therefore, we sought to find out whether there are any correlations between the PS score and any of the personal and clinical history parameters (gender, age, diagnosis, stage, treatment, response, survival, smoker, and number of cigarette packs per year).

### 2.4. PS Score in Healthy Subjects (HC Group)

**Figure 4:**
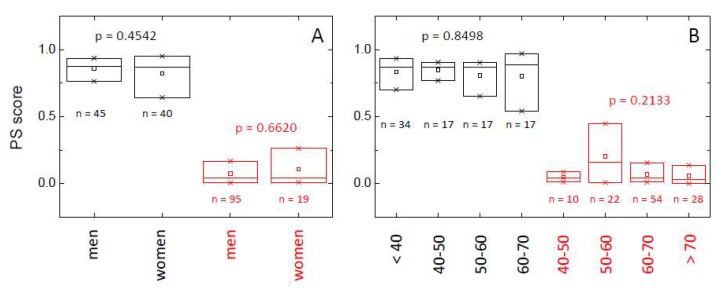
Distribution of the probability score (PS) within healthy individuals (HC group, black) and lung cancer patients (LCP group, red) according to gender (**A**) and age (**B**). The box-plot diagrams indicate Q_1_, Q_2_, and Q_3_, together with the average value (square). The number below each box is the number of subjects within each subgroup. The p-value (Kruskal–Wallis test) indicates there is no statistically significant difference between subcategories (gender and age) within HC and LCP groups (*p* > 0.05).

In order to study the behavior of the proposed PS score, and taking into account its asymmetry for both HC and LCP groups, we have developed a small descriptive analysis using quartiles and the rank-based Kruskal–Wallis test to address if the distribution by group is the same or not. It can be seen (Figure 4, Table S3 and Table 6) there are no significant differences in the distribution of PS score in the HC group according to the gender and age of the individuals (*p*-value > 0.05; Table S3). Therefore, in healthy individuals, the probability of having a normal/altered TLB thermogram does not depend on either gender or age. When looking at the behavior of the classification established by the PS score (i.e., whether PS score > threshold = 0.5 or not), a few subjects (7) are classified as having an altered TLB thermogram (false positives), but they are older than 40, and indeed four of them are older than 50 (Table 6). There is no significant departure from independence between the variables involved.

### 2.5. PS score in Lung Cancer Patients (LCP)

It is clear that the PS score for those individuals with lung cancer (LCP group) is dramatically smaller than the PS score for healthy subjects (HC group), indicating an altered TLB thermogram (Figure 4, Table S3 and Table 6). As it occurred in the HC group, there are no significant differences in the PS score according to gender or age (*p*-value > 0.05; Table S3). However, when looking at the behavior of the classification established by the PS score (i.e., whether PS score > threshold = 0.5 or not), a few subjects (11) were classified as having an unaltered TLB thermogram (false negatives), and most of them are older than 50 (Table 6). There is no significant departure from independence between the variables involved, except for a marginal effect of age in lung cancer patients.

Once it has been established that the PS score is not significantly affected by gender and age, the next step is to explore whether or not the PS score provides information about the stage and particular conditions of the cancer suffered by an individual from the LCP group (i.e., whether it shows some correlation with clinical history information).

**Figure 5:**
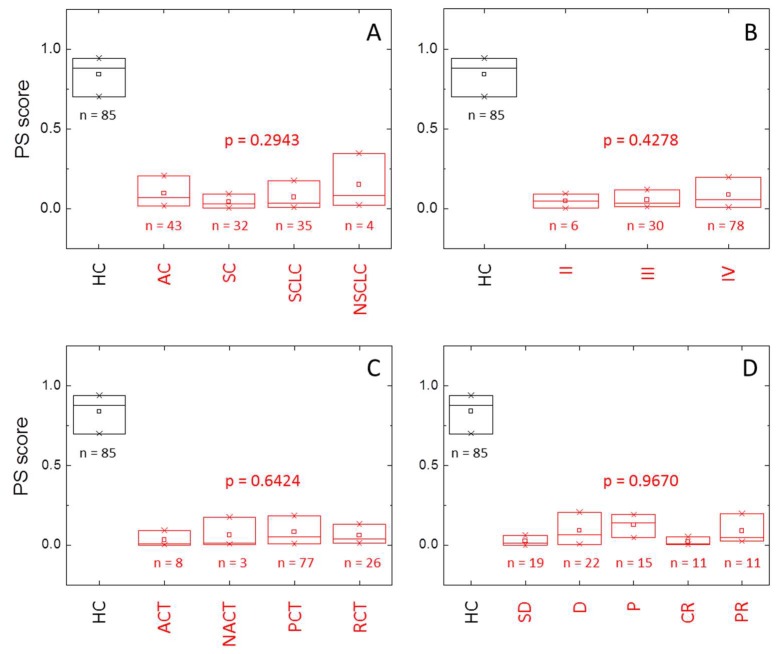
Distribution of the probability score (PS) within healthy individuals (HC group, black) and lung cancer patients (LCP group, red) according to (**A**) diagnostic (AC: adenocarcinoma, SC: squamous cell lung cancer, SCLC: small cell lung cancer, NSLC: other non-small cell lung cancer), (**B**) stage, (**C**) treatment (ACT: adjuvant chemotherapy, NACT: neoadjuvant chemotherapy, PCT: palliative chemotherapy, RCT: radio-chemotherapy), and (**D**) response (SD: stable disease, D: death, P: progression, CR: complete response, PR: partial response). The box-plot diagrams indicate Q_1_, Q_2_, and Q_3_, together with the average value (square). The number below each box is the number of subjects within each subgroup. The *p*-value (Kruskal–Wallis test) indicates there is no statistically significant difference between subcategories (diagnostic, stage, treatment, and response) within HC and LCP groups (*p* > 0.05).

There is no statistically significant relationship between the PS score and diagnostic, tumor stage, treatment, and clinical response (*p* > 0.05) (Table S4). However, tumors diagnosed as squamous or small cell carcinoma show very small PS scores compared to adenocarcinoma or other non-small cell tumors (Figure 5, Table S4). Only a few subjects (11) were classified as having an unaltered TLB thermogram (false negatives), and they mainly correspond to adenocarcinoma (8), and stages III (2) and IV (9) (Table 7). Once again, there is no significant departure from independence between the variables involved.

## 3. Discussion

The utility of TLB thermograms in classifying gastric patients and monitoring melanoma cancer patients has been previously shown [18,19]. For these tasks, two similar approaches based on the deconvolution of serum thermograms into individual temperature-induced transitions according to a phenomenological interpretation of the apparent average excess heat capacity, C_P_, of the serum sample were applied. From that deconvolution, a set of thermogram-derived primary parameters was defined (T_av_, G_1_, A_i_, T_c,i_, w_i_), and they were employed for calculating a second set of secondary or final parameters (AUC_ni_, AP_ni_, Dv_i_). In this work, we have refined the methodology by using appropriate statistical tools for building a model with the ability to classify subjects according to a predictive score: the probability measure of having a healthy/altered serum thermogram, which is expected to correlate with the presence of cancer, as evidenced by previous published work [15,17,18,19].

This model was built using healthy individuals and lung cancer patients, with the final goal of applying the predictive score in clinical practice for estimating the probability of suffering from lung cancer, provided that there would be a strong correlation between an altered serum TLB thermogram and the presence of lung cancer. Therefore, two groups of subjects were employed as a control group (85 healthy individuals, HC) and diseased group (114 lung cancer patients, LCP).

An initial preliminary analysis of the statistical distribution of the 14 individual TLB-derived parameters (T_av_, G_1_, AUC_ni_, AP_ni_, Dv_i_) based on the three quartiles Q_1-3_ suggested that T_av_, G_1_, AUC_n3_, AP_n3_, AUC_n4_, AP_n4_, and Dv_5_ were noticeably different between HC and LCP groups (Table 1, Figure 2). A step forward consisted of performing an ROC analysis (Table 2) from which G_1_, AUC_n3_, AP_n3_, AUC_n5_, and Dv_5_ emerged as statistically relevant parameters. The fact that these two subsets only share some elements indicates that simple analytical approaches will only provide a limited description of the differences between TLB thermograms, and it is an indication of the potential hidden, subtle details within the thermograms.

From a statistical point of view, there exist a range of different techniques that can be used to predict the outcome of a binary random variable. Indeed, most of them are not only useful for making predictions, but they also offer a way of obtaining either a probability or a sort of score for discriminating between the two options. A good overview of these techniques and their use can be found elsewhere [29,30]. While the main features and the motivation of these techniques are quite different, in principle, they are all valid for our goal: elaborating a predictive score for classifying individuals according to their TLB thermograms that correlates with the absence/presence of lung cancer.

The performances of four different predictive tools (GLM, LDA, SVM, and NBC) were compared considering the aforementioned datasets: model 1 based on T_av_, G_1_, AUC_ni_, and AP_ni_; model 2 based on Dv_i_; and model 3 based on the complete set of 14 parameters. GLM outperformed the other three tools when using these three sets of parameters (see Supplementary Materials). Therefore, GLM was selected to construct a probability score (PS) from TLB-derived parameters because it allows the fit of different models to be built and compared based on their parameters and it could be possible to measure the relative importance of TLB-derived parameters to classify diseased/healthy individuals. It is important to bear in mind that the final goal is two-fold: 1) make an appropriate classification of healthy and diseased individuals using TLB; and 2) provide a quantitative score that can be interpreted as the probability for a person to be in a healthy or lung cancer condition according to TLB. Therefore, ROC techniques will not be of interest, as model comparison will not be easy beyond the performance in terms of the classification performance.

The application of GLM revealed different subsets of statistically relevant (*p* < 0.05) TLB-derived parameters for discriminating between HC and LCP groups, depending on the multiparametric models: T_av_, G_1_, and AUC_n3_ for model 1; Dv_3_ and Dv_5_ for model 2; and T_av_, G_1_, AUC_n3_, AUC_n4_, AP_n4_, AUC_n5_, and Dv_4_ for model 3. Interestingly, this subset of relevant TLB parameters partially coincides with those subsets identified with the initial preliminary analysis.

Although somewhat marginally compared to model 1, model 3 provides the best performance using GLM. The PS score obtained with GLM and model 3 results in a 91% success rate or accuracy in properly classifying subjects into healthy and diseased (lung cancer), 90% sensitivity (10% false negative rate), and 92% specificity (8% false positive rate), leading to a remarkable diagnostic odds ratio of 104. Despite the dependency of the confidence intervals (CI) on the sample size, the calculated CI95% values for accuracy, sensitivity, sensitivity, and diagnostic odds ratio are (86%, 95%), (83%, 95%), (84%, 97%), and (39, 280), respectively. These indexes and their CI95%’s support a strong correlation between the PS score and the absence/presence of lung cancer. In fact, the distribution of the PS score for HC and LCP groups is very different and there is a pronounced inter-group difference in the distribution of the PS score for HC and LCP groups: in HC, the median PS score is 0.88, while in LCP, the median PS score is 0.05. The test for the median difference provides a p-value lower than 10^-15^. In addition, the size effect for the binomial classification obtained by the PS score between the two groups can be calculated according to the Cohen d index, which is equivalent to the strictly standardized mean difference, and it is an evaluation index for the discriminating capability of the PS score; using the median values for HC and LCP groups as central tendency measures and the pooled interquartile range as a dispersion measure, a Cohen d index of 3.2 is calculated for the PS score (values larger than 2 are considered highly remarkable).

A good predictive score for clinical practice should not have any bias regarding any factor that is not directly related to the disease it is directed at. Therefore, in our case, the PS score should not show any differences in its distribution according to gender and age within the HC group. However, in the LCP group, there could be some influence of gender or age in the PS score distribution if lung cancer would exhibit specific differential features, depending on gender and age. According to the results (Table S3, Figure 4), there is neither an influence of gender nor age on the PS score in HC and LCP groups. Regarding the lung cancer patients, it would be reasonable to assume that gender should not have any influence on the disease, but there might be a correlation between PS score and age, since it is well-known that age is an important risk factor for cancer. However, the LCP group consists of subjects already suffering from lung cancer, and, therefore, all LCP individuals already show small PS score values. In order to observe a certain correlation between the PS score and age, we should consider a more general set of naïve subjects (i.e., with unknown a priori classification regarding the presence of lung cancer); then, the PS score should show a slight diminishing trend as age increases, being that reduction is dependent on the incidence of cancer along the different age ranges.

On the other hand, it could be reasonable to expect that clinical data of patients (diagnostic, tumor stage, treatment, and response) could have a certain influence on the distribution of the PS score. According to the results (Table S4, Figure 5), the distribution of the PS score shows no dependence on the type of tumor (diagnostic) or on the tumor stage. Very interestingly, although the type of tumor (diagnostic) does not influence the distribution of the PS score, the PS score shows significantly different values for adenocarcinoma and other non-small cell tumors (median values of 0.0672 and 0.0823, respectively), compared to squamous and small cell tumors (median values of 0.0310 and 0.0324, respectively). It would be interesting to find out if the former types of tumor induced larger alterations in serum proteins or metabolites (e.g., increased secretion of metabolites or exosomes), compared to the latter type of tumors. In addition, besides other non-small cell carcinoma, for which there are only four cases in this study, the adenocarcinoma lung cancer presents a higher false negative rate (16%), and this is the lung cancer type with a better prognosis. Squamous and small cell carcinoma, with the second one being a very aggressive lung cancer type, show very low false negative rates (6% and 3%, respectively). Among the few false negative subjects (only 11 individuals, out of 114 in the LCP group, with a PS score higher than 0.5 and, therefore, classified as having an unaltered TLB thermogram), they mainly correspond to adenocarcinoma (8), regarding diagnostic, and stage IV (9), regarding stage. It is important to remember that, as adenocarcinoma is the less aggressive form of lung cancer lesion, it would be expected to produce false negatives because of the low level of serum proteome alteration. However, at this moment, there is no clear connection between tumor stage and alteration of the serum proteome; it might be possible that early tumor stages are highly metabolically active, resulting in a largely markedly altered serum proteome, whereas late stages are less metabolically active, leading to lower levels of serum proteome alterations.

The influence of the treatment applied to the patient or the response was not analyzed because of several reasons: (1) contrary to the diagnostic (type of tumor and stage) that depends on the physiology and natural history of the tumor, the applied treatment depends on the decision of the oncologist based on multiple factors; (2) the response depends on the type and stage of tumor, together with the selected treatment, and, therefore, many interplaying factors are involved; and (3) the patient response to the treatment was only evaluated two months after the first therapeutic intervention, and it could be possible that a two-month interval is not enough to reliably assess and infer valuable and accurate conclusions about the patient evolution. Therefore, a lengthier surveillance time window would be more appropriate, with periodic TLB assessments every 2–3 months. Future studies should include more patients with stage I and II lung cancer, in order to assess whether the stage is correlated with the intensity of TLB serum proteome alterations and the possibility of false negatives, as well as an additional subject group consisting of patients suffering from a chronic non-neoplastic disease as a means to explore and compare potential alterations in serum TLB profiles unrelated to cancer.

## 4. Materials and Methods

### 4.1. Subjects and Samples

Two subject groups, healthy controls (HC group) and lung cancer patients (LCP group), were employed to build the predictive model for lung cancer providing a patient classification score. A comprehensive description of these two groups is shown in the Supplementary Materials.

Consecutive Spanish Caucasian patients with lung cancer (LCP) diagnosed and attended in the Medical Oncology outpatient clinic at the Hospital Clinico Universitario Lozano Blesa in Zaragoza, Spain, from 2015 to 2016, were invited to take part in the study. A total of 114 lung cancer patients were grouped according to their histological type (adenocarcinoma, squamous cell carcinoma, small cell carcinoma, and other different types of non-small cell lung cancer). Moreover, the patients were classified based on different stages at diagnosis (from stage II to IV). Patients with a synchronous history of other malignancies, previous cancer-related treatment, the absence of blood samples, or refusal to participate in the study were considered non-eligible. At the time of inclusion, detailed information was recorded concerning age, gender, smoking habits, tumor-node-metastasis stage (TNM stage) according to the Union for International Cancer Control/American Joint Committee on Cancer (UICC/AJCC 7th Edition) classification, the presence of metastases, the type of treatment, and the histological subtype.

The LCP group was composed of 114 patients diagnosed with lung cancer, with an average age of 64.6 ± 8.7. Most of the patients were men (83% men vs. 17% women) and smokers (64% of the patients were smokers, 29% ex-smokers, and 7% never smoked). Regarding the characteristics of the disease, the majority of the patients were diagnosed at stage IV (68%), followed by stage III (27%), and stage II (5%). All of them were treated according to their stage (68% palliative chemotherapy, 22% chemo-radiotherapy, 7% adjuvant chemotherapy, and 3% neoadjuvant chemotherapy). The response observed in our patients was a 67% controlled disease rate (40% partial response, 10% complete response, and 17% stable disease), 13% progressed disease rate, and 20% death rate after a two-month period. Moreover, the histological distribution is in agreement with the published literature: 37% of adenocarcinomas, followed by 30% of small cell carcinoma, 29% of squamous cell carcinoma, and 4% corresponding to other non-small cell lung cancer).

The HC group consisted of 85 serum samples from gender and age matched Spanish Caucasian subjects, apparently cancer-free, with no previous history of lung cancer, from a FISABIO (Fundacion para el Fomento de la Investigacion Sanitaria y Biomedica de la Comunitat Valenciana) biobank with a homogeneous distribution, including gender (53% men and 47% women), with an average age of 45.2 ± 14.2.

### 4.2. Blood Sample Processing

Approximately 10 mL of peripheral blood from each patient and control subject was collected in serum separator tubes for subsequent TLB analysis. Tubes were gently mixed by immediately after blood collection and centrifuged at 3200 rpm for 10 min (Centrifuge 5702, Eppendorf, Madrid, Spain). Separated plasma was carefully aspirated to avoid hemolysis and contamination of the separated blood phases, and immediately stored in 0.2 mL aliquots at −80 °C with an alphanumeric code for identification until analysis. All participants gave written informed consent to the study protocol, which was previously approved and conducted in accordance with the Ethical Review Board for Clinical Research of the Regional Government (CEIC Aragon). All experimental protocols were approved by CEIC Aragon (Nº CP18/2015, 18/11/2015). All experiments were carried out in accordance with the approved guidelines.

### 4.3. Differential Scanning Calorimetry (DSC)

The heat capacity of serum samples was measured as a function of temperature, C_P_(T), using a high-sensitivity automated differential scanning VP-capillary DSC microcalorimeter (MicroCal-Malvern Panalytical, Malvern, UK). The baseline of the instrument was routinely recorded before experiments. Experiments were performed in diluted serum samples (1:25 in phosphate-buffered saline (PBS)) at a scanning rate of 1 °C/min. Thermograms were baseline-corrected and analyzed using software developed in our laboratory implemented in Origin 7.0 (OriginLab, Northampton, MA, USA). In order to ensure reproducibility, replicate experiments, as well as control experiments with commercial human serum (H4522, Sigma-Aldrich, Barcelona, Spain), were performed periodically.

### 4.4. Thermogram Deconvolution

We have developed a phenomenological model in which the complex thermogram was deconvoluted using six individual transitions modeled with the logistic peak or Hubbert function [18,19]:

(1) $$C_{P}\left(T\right)=C_{P,0}+\overset{i=6}{\underset{i=1}{\sum }}\frac{4A_{i}\exp\left(-\frac{T-T_{c,i}}{w_{i}}\right)}{{\left(1+\exp\left(-\frac{T-T_{c,i}}{w_{i}}\right)\right)}^{2}}$$

where A_i_ is the height of each peak (equivalent to the maximal unfolding heat capacity C_P,max_), T_c,i_ is the center of the peak (equivalent to the mid-transition temperature T_m_), and w_i_ is the width of the peak (C_P_(T_c_ ± w) ≈ 0.8A, and C_P_(T_c_ ± 2w) ≈ 0.4A). The offset parameter C_P,0_ is an adjustable parameter to counterbalance possible errors from baseline correction. For any given serum thermogram, eighteen parameters (A_i_, T_c,i_, and w_i_, for each of the six individual transitions), were obtained after data analysis. These parameters constitute the basic set of parameters from which another final set of parameters (as indicated in the next section) is constructed for the multiparametric comparative quantitative methodology [18,19] aimed at establishing a classification criterion among healthy subjects and lung cancer patients.

### 4.5. Multiparametric Set

The multiparametric method is based on a set of 14 parameters that were defined for the TLB thermogram of each subject in a plasma/protein concentration-independent manner. Therefore, there is no need for protein concentration determination and there are no hidden protein concentration dependencies.

#### 4.5.1. Average temperature, T_av_

This parameter provides the average temperature or first moment of the thermogram when considered as a density distribution function:

(2) $$T_{av}=\frac{\sum_{j}C_{P}\left(T_{j}\right)T_{j}}{\sum_{j}C_{P}\left(T_{j}\right)}$$

where the subscript j runs over the entire range of experimental points in the thermogram.

#### 4.5.2. Skewness, G_1_

This parameter provides a measure of the asymmetry of the thermogram:

(3) $$\begin{array}{c} m_{k}=\frac{\sum_{j}C_{P}\left(T_{j}\right){\left(T_{j}-T_{av}\right)}^{k}}{\sum_{j}C_{P}\left(T_{j}\right)}\\ G_{1}=\frac{m_{3}}{m_{2}^{3/2}} \end{array}$$

#### 4.5.3. Normalized area under the curve, AUC_ni_

This parameter provides the area under the thermogram normalized by any of the peak heights (with i = 2–5):

(4) $$AUC_{ni}=\frac{\sum_{j}C_{P}\left(T_{j}\right)}{A_{i}}$$

#### 4.5.4. Normalized area of the height polygon, AP_ni_

This parameter provides the area of the irregular hexagonal diagram constructed with the heights of the six individual peaks normalized by any of the peak heights (with i = 2–5):

(5) $$AP_{ni}=\overset{s=6}{\underset{s=1}{\sum }}\frac{\sqrt{3}}{4}\frac{A_{s}A_{s+1}}{A_{i}^{2}}$$

#### 4.5.5. Normalized distance value, Dv_i_

This parameter provides the Euclidean distance from the center of the HC group ellipsoid (set of healthy subjects), considering the reference (median) values for the set of healthy subjects, $\bar{T_{av}},\;\bar{AUC_{ni}},\;\bar{AP_{ni}}$:

(6) $$Dv_{i}=\sqrt{{\left(\frac{T_{av}-\bar{T_{av}}}{\bar{T_{av}}}\right)}^{2}+{\left(\frac{AUC_{ni}-\bar{AUC_{ni}}}{\bar{AUC_{ni}}}\right)}^{2}+{\left(\frac{AP_{ni}-\bar{AP_{ni}}}{\bar{AP_{ni}}}\right)}^{2}}$$

### 4.6. Statistical Modeling

The main goal in this work was to build an optimal TLB-derived score exhibiting a strong correlation with a lung cancer condition which could be employed as a clinical tool for lung cancer assessment. That score was to be constructed using the multiparametric dataset introduced above, and three different, but related, predictive models, with each using a different basis set of parameters, were considered: model 1 based on T_av_, G_1_, AUC_ni_, and AP_ni_; model 2 based on Dv_i_; and model 3 based on all 14 parameters.

These three different models were developed to address the problem of constructing a prediction or classification score for lung cancer based on the TLB-derived parameters. In fact, that score will represent the probability of a subject being classified as healthy/diseased according to given TLB-derived parameters (healthy if unaltered TLB thermogram, diseased if altered TLB thermogram). Those models were elaborated using binomial Generalized Linear Models (GLM) with logit link models, since this tool outperformed other statistical tools in discriminating subjects into healthy and diseased (see Results and Supplementary Materials). Because we have to predict a binary variable, the binomial GLM becomes a suitable tool to estimate a logistic regression with the outcome being the probability of absence of lung cancer (i.e., healthy subject) according to a given TLB thermogram.

The only difference between the three GLMs consists of the set of individual TLB-parameters considered: (1) model 1 employs T_av_, G_1_, AUC_ni_, and AP_ni_; (2) model 2 employs Dv_i_; and (3) model 3 employs all 14 parameters. Therefore, in each of the three GLMs, a given set of TLB-derived parameters, {p_k_}, is employed, and the probability score, PS, of an individual with a given thermogram (i.e., with given TLB-derived parameters {p_k_}) being in a healthy condition is given by the following:

(7) $$PS=P\left(\mathrm{healthy}|\left\{p_{k}\right\}\right)=\frac{e^{\mu \left(\left\{p_{k}\right\}\right)}}{1+e^{\mu \left(\left\{p_{k}\right\}\right)}}$$

where µ is a linear function of the TLB-derived parameters:

(8) $$\mu \left(\left\{p_{k}\right\}\right)=a_{0}+\underset{k=1}{\sum }a_{k}p_{k}$$

Additionally, the coefficients {a_k_} are optimally estimated by means of a maximum likelihood estimator, which in turn uses an iterative reweighted least squares algorithm or Newton’s method [29]. This probability can be interpreted straightforwardly as how likely the individual with such a TLB thermogram is free of lung cancer, and, therefore, that probability can be used as a diagnosis criterion. This PS score based on TLB-derived parameters classifies individuals as healthy (negative result, PS > 0.5) and diseased (positive result, PS < 0.5) subjects.

## 5. Conclusions

TLB is a quick, minimally-invasive, low-risk technique that provides real-time, direct information about the proteomic state of blood serum. Through a multiparametric data analysis, the TLB-derived PS score (probability of having an unaltered or healthy TLB serum thermogram) has been elaborated based on well-known statistical methods. In this work, a strong correlation between the PS score and the absence/presence of lung cancer has been demonstrated. In addition, the PS score seems to be an appropriate, robust, unbiased measure of proteomic blood serum alterations because it is not influenced by gender and age, and it provides markedly different values for the probability of having an unaltered/altered serum proteome for healthy subjects and lung cancer patients. The strong correlation between PS score and lung cancer translates into a 91% success rate in classifying subjects, 8% false positive rate, and 10% false negative rate, with a diagnostic odds ratio of 104. Therefore, TLB is a new type of liquid biopsy based on serum thermograms that may be employed as a diagnosis and prognosis tool in cancer management.

## Figures and Tables

**Table 1:** Quartiles of individual thermal liquid biopsy (TLB)-derived parameters in healthy control (HC) and lung cancer patient (LCP) groups.

Group	HC Group			LCP Group		
Parameter	Q1	Q2	Q3	Q1	Q2	Q3
T_av_	67.59	67.95	68.39	67.78	68.16	68.71
G_1_	0.1333	0.2588	0.3521	0.2899	0.4436	0.5662
AUC_n2_	19.86	26.89	43.66	18.72	24.20	36.15
AP_n2_	0.6233	1.127	2.762	0.4884	0.9303	1.902
AUC_n3_	20.39	22.76	25.94	21.59	25.76	33.09
AP_n3_	0.5914	0.8038	1.016	0.7416	0.9723	1.551
AUC_n4_	28.69	39.55	51.97	25.74	33.30	46.76
AP_n4_	1.457	2.416	4.028	0.9898	1.619	3.508
AUC_n5_	39.20	43.20	50.93	36.73	47.69	63.12
AP_n5_	2.265	2.876	4.148	2.150	3.120	6.294
Dv_2_	1.057	1.190	1.867	1.094	1.204	1.393
Dv_3_	1.008	1.043	1.121	1.014	1.072	1.509
Dv_4_	1.043	1.173	1.375	1.081	1.184	1.3333
Dv_5_	1.003	1.035	1.160	1.025	1.197	1.480

**Table 2:** Receiver Operating Characteristic (ROC) analysis for individual thermal liquid biopsy (TLB)-derived parameters.

Parameter	Success Rate	Sensitivity	Specificity	Threshold	Trend
T_av_	57.8	61.4	52.9	68.0	↓
G_1_	71.4	63.2	82.3	0.37	↓
AUC_n2_	53.8	48.2	61.2	23.9	↑
AP_n2_	56.8	62.3	49.4	1.23	↑
AUC_n3_	63.8	59.7	69.4	24.9	↓
AP_n3_	61.8	64.0	58.8	0.88	↓
AUC_n4_	58.8	53.5	65.9	34.0	↑
AP_n4_	59.3	54.4	65.9	1.82	↑
AUC_n5_	61.3	57.9	65.9	45.4	↓
AP_n5_	58.3	43.9	77.6	4.26	↓
Dv_2_	53.3	58.8	45.9	1.17	↓
Dv_3_	58.8	41.2	82.4	1.14	↓
Dv_4_	56.3	65.8	44.5	1.11	↓
Dv_5_	63.3	59.7	68.2	1.08	↓

**Note:** ROC analysis using the Youden method allowed the threshold to be calculated for any individual parameter for classifying subjects as healthy (negative, unaltered TLB thermogram) and diseased (positive, altered TLB thermogram). The trend symbol indicates the direction for classification as a healthy subject: ↑↓ means that, in general, subjects classified as healthy (negative result according to TLB) showed values that were larger/smaller than the indicated threshold.

**Table 3:** Summary of the application of Binomial Generalized Linear Model with Logistic Regression (GLM) to the three models.

Model	Parameter	*z*-Value	*p*-Value
Model 1	T_av_	−5.14	0.00000
	G_1_	−6.38	0.00000
	AUC_n2_	−1.18	0.23689
	AP_n2_	0.931	0.35192
	AUC_n3_	−2.59	0.00949
	AP_n3_	0.975	0.32937
	AUC_n4_	1.38	0.16735
	AP_n4_	−1.28	0.20197
	AUC_n5_	1.36	0.17247
	AP_n5_	1.46	0.14450
Model 2	Dv_2_	1.29	0.19585
	Dv_3_	−2.14	0.03265
	Dv_4_	−1.22	0.22283
	Dv_5_	−1.96	0.04993
Model 3	T_av_	−4.63007	0.00000
	G_1_	−6.30023	0.00000
	AUC_n2_	−1.45263	0.14633
	AP_n2_	1.68493	0.09200
	AUC_n3_	−2.84924	0.00438
	AP_n3_	1.05694	0.29054
	AUC_n4_	2.80208	0.00508
	AP_n4_	−2.36746	0.01791
	AUC_n5_	2.15199	0.03140
	AP_n5_	−1.31683	0.18789
	Dv_2_	−1.65683	0.09755
	Dv_3_	−0.70715	0.47947
	Dv_4_	−2.26054	0.02379
	Dv_5_	0.04718	0.96237

**Table 4:** Model comparison based on the likelihood ratio and on Akaike and Bayesian information criteria.

Indexes	Model 1	Model 2	Model 3
Degrees of Freedom	−4	−10	n.a.
Residual Degrees of Freedom	188	194	184
Residual Deviance	127	241	118
Equivalency with model 3 P(>χ^2^)	0.06269	0.00000	n.a.
Akaike Information Criterion	149	251	148
Bayesian Information Criterion	185	267	198

n.a.: not applicable.

**Table 5:** Model comparison based on the ability to classify subjects.

**Model**	**Success Rate**	**Sensitivity**	**Specificity**
Model 1	88	89	87
Model 2	69	73	65
Model 3	91	90	92
**LOO Test**	**Success Rate**	**Sensitivity**	**Specificity**
Model 1	86	86	87
Model 2	65	60	68
Model 3	87	86	88

**Table 6:** Contingency tables for gender and age (probability score (PS) threshold = 0.5).

**Group**	**Gender**	**PS > 0.5** **True Negatives**	**PS < 0.5** **False Positives**	***p*-Value**
Healthy Controls	Men	43 (96%)	2 (4%)	0.2460
	Women	35 (88%)	5 (12%)	
	**Gender**	**PS > 0.5** **False Negatives**	**PS < 0.5** **True Positives**	***p*-Value**
Lung Cancer Patients	Men	8 (8%)	87 (92%)	0.2394
	Women	3 (16%)	16 (84%)	
	**Age**	**PS > 0.5** **True Negatives**	**PS < 0.5** **False positives**	***p*-Value**
Healthy Controls	< 40	32 (94%)	2 (6%)	0.3894
	40–50	17 (100%)	0 (0%)	
	50–60	14 (82%)	3 (18%)	
	60–70	15 (88%)	2 (2%)	
	**Age**	**PS > 0.5** **False Negatives**	**PS < 0.5** **True Positives**	***p*-Value**
Lung Cancer Patients	40–50	1 (10%)	9 (90%)	0.04748
	50–60	5 (23%)	17 (77%)	
	60–70	5 (9%)	49 (91%)	
	70–iii	0	28 (100%)	

**Note:***p*-Values were calculated according to Fisher’s independence test.

**Table 7:** Contingency table for clinical history information (probability score (PS) threshold = 0.5).

**Group**	**Diagnostic**	**PS > 0.5** **False Negatives**	**PS < 0.5** **True Positives**	***p*-Value**
Lung Cancer Patients	Adenocarcinoma	7 (16%)	36 (84%)	0.1294
	Squamous cell carcinoma	2 (6%)	30 (93%)	
	Small cell carcinoma	1 (3%)	34 (97%)	
	Other non-small cell carcinoma	1 (25%)	3 (75%)	
	**Stage**	**PS > 0.5** **False Negatives**	**PS < 0.5** **True Positives**	***p*-Value**
Lung Cancer Patients	II	0 (0%)	6 (100%)	0.8541
	III	2 (7%)	28 (93%)	
	IV	9 (12%)	69 (88%)	

Note: *p*-Values were calculated according to Fisher’s independence test.

## Supplementary Materials

The following are available online at https://www.mdpi.com/2072-6694/11/7/1012/s1: Figure S1: Graphical description of the HC (healthy controls) and LCP (lung cancer patients) groups; Table S1: Main descriptive measures for the 14 TLB-derived parameters, Table S2: Comparative study of the performance of GLM, LDA, SVM, and NBC in the three models, Table S3: PS score vs. gender and age, Table S4: PS score vs clinical history information.

## Abbreviations

CI95%	confidence interval at 95% significance level
DSC	differential scanning calorimetry
ECM	extracellular matrix
GLM	binomial generalized linear model with logistic regression
HC	healthy control group
LB	liquid biopsy
LCP	lung cancer patients group
LDA	linear discrimination analysis
LDCT	low-dose computed tomography
NBC	naïve Bayes classifier
PBS	phosphate-buffered saline
Q	quartile
ROC	receiver operating characteristic
SVM	support-vector machine for classification
TLB	thermal liquid biopsy
