# Supplementary material for: Thermal Liquid Biopsy (TLB): A Predictive Score Derived from Serum Thermograms as a Clinical Tool for Screening Lung Cancer Patients
Source: Cancers (Basel). 2019 Jul 19;11(7):1012. doi: 10.3390/cancers11071012 (PMC6678750; doi:10.3390/cancers11071012)
Supplement: Supplementary file 1 [file cancers-11-01012-s001.pdf]

Supplementary Materials: Thermal Liquid Biopsy (TLB): A predictive score derived from serum thermograms as a clinical tool for screening lung cancer patients

Alberto Rodrigo, Jorge L. Ojeda, Sonia Vega, Oscar Sanchez-Gracia, Angel Lanas, Dolores Isla, Adrian Velazquez-Campoy and Olga Abian

Table S1. Main descriptive measures for the 14 TLB-derived parameters.

HC Group	Minimum	Q1	Q2	Average	Q3	Maximum
T_{av}	65.93	67.59	67.95	67.98	68.39	69.08
G_1	-0.3757	0.1333	0.2588	0.2330	0.3521	0.7714
AUC_{n2}	7.011	19.86	26.89	36.92	43.66	201.7
AP_{n2}	0.2438	0.6233	1.127	3.598	2.762	81.29
AUC_{n3}	14.98	20.39	22.76	24.02	25.94	76.84
AP_{n3}	0.2934	0.5914	0.8038	0.9215	1.016	4.343
AUC_{n4}	2.882	28.69	39.55	43.90	51.97	148.2
AP_{n4}	0.07751	1.457	2.416	3.232	4.028	16.24
AUC_{n5}	22.06	39.20	43.20	49.67	50.93	143.8
AP_{n5}	0.2949	2.265	2.876	4.203	4.148	33.44
Dv_2	0.9857	1.057	1.190	3.244	1.867	71.43
Dv_3	0.9855	1.008	1.043	1.175	1.121	5.101
Dv_4	0.9861	1.043	1.173	1.443	1.375	6.069
Dv_5	0.9825	1.003	1.035	1.377	1.160	10.93

LCP Group	Minimum	Q1	Q2	Average	Q3	Maximum
T_{av}	65.04	67.79	68.16	68.23	68.71	70.63
G_1	-0.1058	0.2899	0.4436	0.4518	0.5662	3.622
AUC_{n2}	7.011	18.72	24.20	29.82	36.15	98.32
AP_{n2}	0.0952	0.4884	0.9303	1.924	1.902	20.61
AUC_{n3}	8.830	21.59	25.76	34.63	33.09	263.71
AP_{n3}	0.2049	0.7416	0.9723	2.145	1.551	29.33
AUC_{n4}	4.842	25.74	33.30	122.7	46.76	8098.0
AP_{n4}	0.2855	0.9898	1.619	261.2	3.508	28790
AUC_{n5}	18.95	36.73	47.69	101.8	63.12	1858
AP_{n5}	0.1778	2.150	3.120	152.3	6.294	8070
Dv_2	0.9857	1.094	1.204	1.909	1.393	17.32

Dv_3	0.9855	1.014	1.072	2.562	1.509	35.50
Dv_4	0.9861	1.081	1.184	3.872	1.333	149.7
Dv_5	0.9825	1.025	1.197	3.644	1.480	85.04

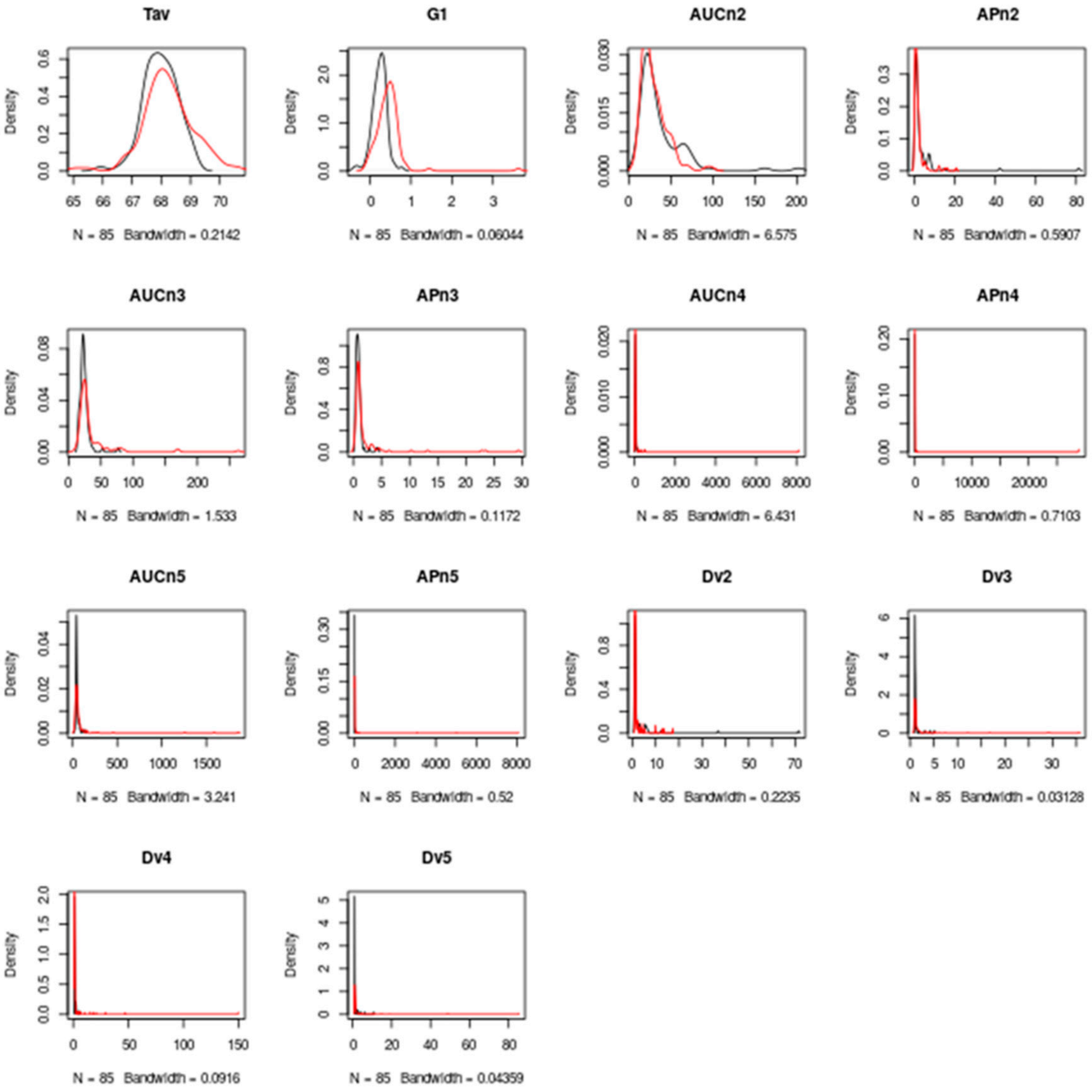

Figure S1. Non-parametric density estimators for the 14 individual TLB-derived parameters. HC group (black) / LCP group (red).

Table S2. Comparative study of the performance of GLM, LDA, SVM, and NBC on the three models.

	Parameter	Success rate	Sensitivity	Specificity
**Model 1**	GLM	88	89	87
	LDA	84	88	79
	SVM	90	87	95
	NBC	55	24	98

**Model 2**	**Parameter**	**Success rate**	**Sensitivity**	**Specificity**

	GLM	69	73	65
	LDA	58	96	6
	SVM	61	97	13
	NBC	55	25	96

	**Parameter**	**Success rate**	**Sensitivity**	**Specificity**
**Model 3**	GLM	91	90	92
	LDA	85	88	81
	SVM	90	88	96
	NBC	56	25	96

Four statistical tools (GLM, LDA, SVM, and NBC) were employed for classifying subjects into healthy and diseased individuals using the three models: 1) model 1 with T_{av}, G_1, AUC_{ni}, and AP_{ni}; 2) model 2 with Dv_i; and 3) model 3 with all 14 parameters.

Table S3. PS score vs gender & age (*p*-value from Kruskal-Wallis test).

	**Healthy Controls**					**Lung Cancer Patients**				
**Gender**	**N**	**Q1**	**Q2**	**Q3**	***p*-Value**	**N**	**Q1**	Q2	Q3	*p*-Value
Men	45	0.7649	0.8776	0.9380	0.4542	95	0.0075	0.0450	0.1681	0.6620
Women	40	0.6448	0.8722	0.9515		19	0.0101	0.0457	0.2632	

	**Healthy Controls**					**Lung Cancer Patients**				
**Age**	N	**Q1**	**Q2**	**Q3**	***p*-Value**	**N**	**Q1**	Q2	Q3	***p*-Value**
< 40	34	0.7022	0.8699	0.9343	0.8498	0				0.2133
40 - 50	17	0.7706	0.8692	0.9062		10	0.0120	0.0443	0.0879	
50 - 60	17	0.6528	0.8707	0.9035		22	0.0090	0.1574	0.4478	
60 - 70	17	0.5411	0.8883	0.9713		54	0.0114	0.0423	0.1564	
70 -	0					28	0.0003	0.0309	0.1375	

Table S4. PS score vs. clinical history information (p-value from Kruskal-Wallis test).

	**Lung Cancer Patients**				
**Diagnostic**	**N**	**Q1**	**Q2**	Q3	***p*-Value**
Adenocarcinoma	43	0.0166	0.0672	0.2046	0.2943
Squamous cell carcinoma	32	0.0040	0.0301	0.0899	
Small cell carcinoma	35	0.0078	0.0324	0.1739	
Other non-small cell carcinoma	4	0.0223	0.0823	0.3456	

	**Lung Cancer Patients**				
**Stage**	**N**	**Q1**	**Q2**	Q3	***p*-Value**
II	6	0.0008	0.0456	0.0923	0.4278
III	30	0.0096	0.0334	0.1169	
IV	78	0.0079	0.0566	0.1964	

	**Lung Cancer Patients**				
**Treatment**	**N**	**Q1**	**Q2**	Q3	***p*-Value**

Adjuvant Chemotherapy	8	0.0018	0.0072	0.0917	0.6424
Neoadjuvant Chemotherapy	3	0.0061	0.0113	0.1764	
Palliative Chemotherapy	77	0.0078	0.0546	0.1845	
Radio-Chemotherapy	26	0.0127	0.0412	0.1327	

			**Lung Cancer Patients**		
**Response**	**N**	**Q1**	**Q2**	Q3	***p*-Value**
Stable disease	19	0.0004	0.0133	0.0618	0.0967
Death	22	0.0065	0.0648	0.2063	
Disease progression	15	0.0468	0.1419	0.1915	
Complete response	11	0.0038	0.0091	0.0518	
Partial response	46	0.0246	0.0469	0.1981	
